# Exploring the Use of Little Cigars by Students at a Historically Black University

**Published:** 2008-06-15

**Authors:** David H Jolly

**Affiliations:** Department of Public Health Education, North Carolina Central University

## Abstract

**Introduction:**

Considerable evidence exists that little cigars are popular among African American adolescents and young adults who smoke. However, few studies have been published on the use of this tobacco product by young blacks in the United States. This research investigated little-cigar use among students at a historically black university in the southeastern United States.

**Methods:**

As a follow-up to a survey on tobacco use among freshmen that revealed unexpectedly high rates of little-cigar use, 3 focus groups were conducted with current or former smokers of little cigars. Topics included preferred brands of little cigars, preference for little cigars over cigarettes, social contexts for smoking little cigars, perceived health risks of smoking little cigars relative to smoking cigarettes, and thoughts about quitting.

**Results:**

Focus group participants preferred little cigars to cigarettes for various reasons, among them taste, smell, a better "buzz," social purposes, status, and perceptions that smoking little cigars is less addictive and less harmful than smoking cigarettes. Opinions on health risks varied; some participants believed that health risks can be reduced by removing the inner liner of little cigars.

**Conclusion:**

Use of little cigars should be addressed in tobacco research, use prevention, and use cessation efforts, targeting students at historically black colleges and perhaps other young African Americans. Results also suggest that clear distinctions should be made among cigarettes, little cigars, and cigars, and that tobacco use prevention and cessation programs should debunk myths that little cigars are a safe alternative to cigarettes. Study findings should be confirmed and elucidated through additional research.

## Introduction

Evidence exists in both scientific literature (1-3) and the media (4) that little cigars are popular among African American adolescents and young adults who smoke. However, few studies have been published in tobacco research literature on this form of tobacco or its use by young blacks in the United States (1,2,5).

African Americans aged 18 to 24 years are less likely to smoke tobacco than their white contemporaries (6,7). Nevertheless, college-aged African Americans should be considered a priority population in tobacco control efforts for several reasons. First, African American smokers start using tobacco later than white smokers, often after high school (8,9), and, as adults, smoke cigarettes at about the same rate (10). Second, blacks are disproportionately affected by smoking-related diseases (11). Third, young blacks are targeted heavily by the tobacco industry. Tobacco companies have long marketed their products to young African Americans (12-14); recent promotions such as Brown & Williamson's Kool Mixx campaign suggest no inclination to cease these efforts (15,16).

Smoking behaviors include choosing tobacco products. This paper focuses on the use of little cigars (e.g., Black & Milds, Swisher Sweets). Also known as *cigarillos*, little cigars are slightly larger than cigarettes but much smaller than traditional full-sized cigars. Some (e.g., Black & Milds) are filled with pipe tobacco. In recent years, manufacturers have introduced flavored little cigars, marketing them heavily to young adults. Little cigars are generally sold in packages of 5. In North Carolina in 2007, a pack cost approximately $2.50, and singles were sold for $0.75. Between 1990 and 2005, annual taxable sales of cigarettes in the United States declined 31%, but during the same period sales of little cigars doubled (17).

The health risks associated with smoking cigars are well documented (18-22) and include coronary heart disease, chronic obstructive pulmonary disease, and cancers of the oral cavity, larynx, esophagus, and lung. In addition to health concerns, cigars pose the risk of addiction to nicotine. Both full-sized cigars and little cigars contain considerable amounts of nicotine. Although total nicotine content of little cigars differs from product to product (23), one report suggests that, compared with cigarettes, little cigars can deliver between 2 and 5 times the nicotine per gram of tobacco (24).

A literature review revealed only a few published studies that specifically address the use of little cigars. Malone et al (1) conducted focus groups with African American adolescents aged 14 to 18 years about little-cigar use. They found that many youths were smoking little cigars without identifying the product as either cigar or cigarette, and the youths often viewed little cigars as being safer than both. Page and Evans (2) revealed a similar phenomenon in their ethnographic observations and interviews with black adolescents aged 11 to 15 years.

Richter et al (5) used focus groups to explore young adult smokers' perceptions of the health risks of cigarettes and nontraditional tobacco products, including little cigars. Compared with black participants, Hispanic and non-Hispanic white participants were more likely to rate little cigars as more harmful than their preferred cigarette. Black participants were more likely than other participants to view all tobacco products as equally risky or to rate little cigars as safer than their preferred cigarette.

In one of the few studies to explore why youth use cigars, Soldz and Dorsey (25) conducted a survey of middle and high school students and found that current cigar users were much more likely than nonusers to agree with the statements that cigars "taste good," "smell good," and are "something different to try" than the statements that cigars "are not as bad for you as cigarettes" or "give you a good buzz." Unfortunately, this study did not differentiate between full-sized cigars and little cigars, and whether these findings pertain to African Americans is unclear, as data were not analyzed by race and only 2.1% of respondents were black.

The largest exploration of young people's patterns of cigar use and their perceptions of cigar health risk was conducted by the Office of the Inspector General (26). The study involved a survey and focus groups with 230 urban (i.e., predominantly black) and suburban (i.e., predominantly white) teenagers. Most teenagers were in high school or middle school, and one third were current smokers of cigars. Cigar use in general and little-cigar use in particular were higher among the urban students than among the suburban students. Cigar smokers noted that they typically smoked at parties, often when they were drinking alcohol, and although they knew the general risks of tobacco use, they were much less aware of the specific risks of smoking cigars. Most cigar smokers agreed that regular cigar smoking was addictive, but many were uncertain. Unfortunately, findings from the focus groups did not distinguish between urban and suburban youth, black and white youth, or full-sized and little cigars.

The research described here was conducted among students at a historically black university in the southeastern United States. Focus groups of current or former smokers of little cigars were used to explore reasons for using this product, the contexts of that use, and perceived health risks of smoking little cigars.

## Methods

In fall 2003, a brief paper-and-pencil, self-administered survey on tobacco use was conducted in sections of a required freshman course at a historically black university. The survey revealed unexpectedly high rates of little-cigar use. Of the 684 students (219 male and 465 female) who completed the survey, 25% (63 male students and 107 female students) reported smoking tobacco in the past 30 days. Asked what products they had smoked in the past month, students reported little cigars much more often than cigarettes (74% vs 44%). The preference for little cigars over cigarettes was stronger among male students than among female students but held for both groups. Of male smokers, 84% reported smoking little cigars (either exclusively or in combination with cigarettes), and only 33% smoked cigarettes (either exclusively or in combination with little cigars). The comparable figures among female smokers were 69% for little cigars and 50% for cigarettes.

To explore these findings further, 3 focus groups were conducted in July 2005. Using handbills that briefly described the focus groups, 2 student interns directly recruited participants by approaching students at gathering places on campus and asking them if they currently smoke or formerly smoked little cigars. Those students who answered affirmatively were invited to participate in the focus groups. The only other eligibility criteria were being African American and being enrolled at the university.

The author conducted 2 focus groups and was present for the third, which was facilitated by an intern. The groups lasted 60 to 90 minutes, and participants received a $30 gift certificate for their time. The groups were not segmented in any way; students simply chose the group that was most convenient for them. The author developed a set of focus group questions in consultation with health education students at the university, some of whom had themselves smoked little cigars. Topics included participants' current smoking status, brands of little cigars smoked, frequency of smoking and amount smoked, reasons for smoking little cigars instead of cigarettes, perceived risks relative to cigarettes (in terms of both addiction and disease), intentions to quit, and the social context for smoking little cigars. (Responses to questions about quitting are not reported in this paper.)

Written notes were taken during each group. With participants' permission, the discussions were also tape-recorded, and the recordings were transcribed verbatim. The author reviewed the tapes and analyzed original notes and transcriptions to identify important issues and recurring themes. Given the small number of focus groups conducted, the author coded text of the original notes and transcriptions by hand, highlighting segments of text reflecting identified issues and themes.

The focus group protocol (as well as the earlier survey) was approved by the institutional review board of the university where the research was conducted.

## Results

Three focus groups were held, and 21 African American students, 17 male and 4 female, participated. Ages ranged from 19 to 25 years. Of participants, 20 were current smokers of little cigars; one was a former user. Only 6 of the 21 also smoked cigarettes; one other was a former cigarette smoker. Black & Milds (often referred to simply as Blacks) were overwhelmingly the brand of choice for little cigars. In fact, for these students the name *Blacks* was synonymous with little cigars.

### Why little cigars are preferred to cigarettes

When asked why they preferred little cigars, participants provided various responses.

***Taste and smell.*** A common response was that little cigars taste better than cigarettes. Several students noted that little cigars have a "smoother" taste, and one student said, "A cigarette tastes like chemicals." Black & Milds are filled with pipe tobacco, and generally participants found the odor pleasing, especially compared with that of cigarettes. As one student put it, "Cigarettes stink . . . [but] a Black smells so good." Some participants were concerned about others' reactions to the smell of cigarettes, especially members of the opposite sex.

***"Buzz."*** Many students reported that they smoked tobacco for the feeling it gave them and that the feeling from little cigars was better or more intense compared with the feeling from cigarettes. One student remarked, ". . . the first cigarette you also get a buzz, but after that there is nothing but cigarette. But with Black & Milds, like you get one every time."

***Social purposes.*** According to many participants, smoking little cigars was an integral part of their social life, especially on occasions involving alcohol (e.g., at parties, at clubs). Typical comments included, "I think drinking and smoking Blacks goes together" and "What would happen is you were drinking and smoking, and it just went pretty well hand in hand." Many students noted that in a social setting a little cigar is often passed among friends, much like a marijuana joint. "Normally people smoke and pass," said one participant. "We might share while in the line at the club," added another.

***Status.*** Several participants expressed the opinion that many young people had taken up smoking little cigars because it is in vogue. One participant observed, "Some . . . just want to look cool in the club." During one focus group a student intern in this project noted that many popular hip-hop and rap artists smoke little cigars in music videos. He and several participants expressed opinions that these celebrities may create or reinforce the "cool" image of little cigars.

***Beliefs related to addiction and harm.*** Many participants viewed little cigars as being less addictive and less harmful to their health compared with cigarettes. These reasons are explored in detail later.

Two reasons unrelated to cigarettes were also offered for smoking little cigars:

***Stress.*** Many participants mentioned using little cigars to cope with stress. One participant said, "After class, after work, after an argument . . . I will smoke a Black to calm down." Reported another, "I work in customer service. . . . When I go home . . . I just sit back and smoke a Black and reflect on my day, look at who I was about to cuss out."

***Marijuana use.*** A few students said they smoked little cigars in conjunction with marijuana to heighten the effects of the marijuana. One participant remarked, "Now I smoke after weed to get an extra boost."

### How and how much little cigars are smoked

Participants indicated that inhaling was necessary to get a buzz from little cigars, and almost all participants reported doing so. One participant said not inhaling "is a waste of money."

When participants were asked how many little cigars they smoked, their responses ranged from 1 pack (i.e., 5 cigars) per day to 1 pack every 2 weeks. Those participants who also smoked cigarettes indicated that they smoked more cigarettes than little cigars. One student noted that he smoked 3 packs of cigarettes per week but only 1 pack of Blacks per week.

Many participants indicated that they did not smoke an entire little cigar in a single session. Said one, "I will hype me a Black in the morning, smoke a little before I go to class, then go to class and come back out, and I smoke a little bit more. And then I might go home . . . and smoke the rest of the Black."

Other students stressed that they do not smoke a whole little cigar by themselves, referring to the practice of passing a little cigar among friends. A participant stated, "I can't smoke a whole one by myself. . . . Like I said, I got to smoke with one of my boys." Another participant commented, "Collectively we might smoke five to six Blacks in a day." Summing up this discussion, a student commented, "You got to share the Black."

### Beliefs about addiction

The belief that little cigars are not as addictive as cigarettes was widespread. A participant remarked, "In cigarettes there are more things to get addicted to." Another participant observed, "You see people smoking cigarettes; they be outside in like 10-degree weather, because they need a cigarette. . . . If I want a Black and even if I want it . . . bad, I still won't do anything like [that]." A third participant simply stated, "I could shake a Black, but I couldn't shake cigarettes."

A minority of students disagreed. One declared, "Black & Milds in some cases [are] just as addictive — different gun, same bullet." Another student noted that he had started smoking little cigars to stop smoking cigarettes. He said it worked, but now he felt addicted to little cigars. Even so, he was confident he could stop but said he had not tried: "I don't feel that I smoke enough Blacks in a day for it to be a serious health risk to me."

### Beliefs about health risks

Participants' opinions were divided regarding the health risks of smoking little cigars compared with the risks of smoking cigarettes; some participants believed little cigars were less harmful than cigarettes. Several students noted that little cigars are not addressed in antismoking messages. One student remarked, "I ain't never heard anything about Black & Milds being bad . . . but you hear everything about cigarettes and how they give you cancer and all." Other students claimed a difference in ingredients: "[There are] more harmful things in cigarettes that are not in Blacks, like more gas, toxic gases, and stuff like that." Still other participants cited a difference in the quantity consumed. One student stated, "I think that it is not as bad, because you don't smoke Black & Milds as much, at least I don't."

Other students expressed the opinion that the risks of cigarettes and little cigars are equivalent. "It's the same thing; tobacco is tobacco," said one student. In a more nuanced observation, another student commented, "Smoking a Black you might get the same disease as that cigarette smoker is going to get later on, but you are going to get it years after them."

Still other participants thought little cigars are more harmful than cigarettes and offered different explanations. Some students suggested that little cigars are a stronger tobacco product. For example, one student remarked, "One Black & Mild is just like smoking 10 cigarettes or something like that."

Several students expressed the opinion that toxic chemicals are concentrated in a paper liner in Black & Milds and that this paper makes little cigars more harmful than cigarettes. They referred to this liner as the "cancer stick." These students believed that the risk of smoking little cigars can be reduced by removing the liner through a process called *hyping*, also known as *freaking* (2). As they described it, hyping involves taking the plastic tip off the cigar, completely emptying the tobacco from the wrapper, removing the paper lining inside the wrapper, and returning the tobacco to the wrapper. The primary purpose of hyping is to improve the smoking experience (i.e., making it easier to inhale deeply and "get a good buzz"), but because of students' beliefs that the liner contains high levels of harmful chemicals, some students thought removing it also made little cigars safer.

Confusion about the purpose of the paper liner was considerable. One participant remarked, "My brother [says the] cancer stick is like the filter. I said, nah, the cancer stick can't be no filter, but I felt like that little stick was putting more harm in my body than good and if I take it out, at least I will be winning a little bit."

Several students expressed interest in learning more about little cigars and their risks. One stated, "I want to find out what is in a Black. I have been smoking this for years, and I am going to find out what is really in it." In one focus group, the facilitator asked if participants would want to read factual information about little cigars. All members of the group agreed they would.

## Discussion

Published research on the use of little cigars by young African Americans is scant. A literature review identified 3 studies (1,2,5), 2 of which (1,2) focused on minority adolescents younger than the target population in this study. Other studies (3,25,26) looked at cigar use by young people but did not consistently distinguish between black and white youth or between full-sized and little cigars. Nevertheless, some themes that emerged in this study were similar to those documented earlier. These themes include the widespread popularity of Black & Milds (1-3,5,26), the practices of sharing, hyping, and using little cigars to boost the effects of marijuana (1,2), taste and smell as primary reasons for smoking cigars (5,25,26), and confusion about the risks of smoking cigars compared with cigarettes (1,2,5,26).

Among the most disturbing findings in this study are those regarding perceived health risks of little cigars. As in previous studies (1,2,5,26), this study identified beliefs among some participants that little cigars are less addictive and pose fewer health risks than cigarettes. Some students cited these beliefs as the reason they preferred little cigars to cigarettes. However, these beliefs were far from universal; many participants expressed the opinion that smoking little cigars was as dangerous as smoking cigarettes, if not more dangerous.

One factor contributing to the potential harm from little cigars is the way they are smoked. In general, smoke from neither cigars nor pipes is directly inhaled. This presumably explains why rates of respiratory diseases are significantly lower among cigar and pipe smokers than among cigarette smokers, although rates are still substantially higher among cigar and pipe smokers than among nonsmokers (18,19). However, almost all members of the focus groups reported inhaling little cigars, primarily to get the "buzz" that many said made smoking little cigars pleasurable. These students may think little cigars are safer because they are not cigarettes, but they are inhaling them as if they were cigarettes.

Richter et al (5) have asked whether young adult users of nontraditional tobacco products like little cigars believe the health risks of these products are the same as with cigarettes and simply choose to ignore those risks, or whether they are truly unaware of the risks because they view nontraditional products as being very different products from cigarettes. Their own findings suggest that the former may be the case for African American young adults, but Page and Evans' study (2) indicates that the latter may be true for young African American youth. They interviewed adolescents aged 11 to 15 years who did not identify little cigars as being either cigarettes or cigars and some adolescents who did not even associate little cigars with tobacco. The study reported here included many participants who seemed to be ignoring perceived health risks, some participants who were unaware of the health risks, and other participants who expressed confusion about the health risks.

Some college students in the current study offered logical, if incorrect, explanations for why they believe that little cigars pose fewer health risks than cigarettes (e.g., little cigars are not mentioned in public health antismoking messages, little cigars have fewer chemicals and contain less nicotine than cigarettes). Some students played down the health risks by noting that they smoke fewer little cigars than cigarette smokers smoke cigarettes. Other students seemed to engage in "magical thinking" to justify their use of little cigars. One student reasoned that smoking little cigars must be less harmful than smoking cigarettes, because when he smoked a little cigar, the plastic tip did not turn brown like the filter of a cigarette does. Another student rationalized her use of little cigars by saying her grandmother smoked cigarettes and died of a smoking-related disease.

Devising such creative rationales is easy when there is genuine confusion about risk and when hard facts are unavailable to clear up the confusion. Many participants expressed such uncertainty as well as an interest in learning more about little cigars. However, few antitobacco materials specifically address little cigars. The findings here suggest that African American college students might be more attentive to factual information about little cigars than they are to comparable information about cigarettes, which they are likely to have encountered before and which they may see as irrelevant to their smoking behaviors. The probability that students would pay attention to such information might increase if the information reflected the cultural context in which students consume little cigars. Brown & Williamson has exploited hip-hop culture to market Kool cigarettes to young African Americans (15,16). Advocates of tobacco use prevention and cessation could employ similar strategies in the social marketing of messages about little cigars.

The focus groups confirmed the finding from earlier studies (2,26,27) that language is critical when addressing tobacco issues with young African Americans — both adolescents and young adults. First, it cannot be assumed that smoking implies the use of tobacco, because many participants in this and other studies (1,2,26) also reported smoking marijuana. Second, clarifying what is meant by little cigars is important. Some participants were unclear that Black & Milds were little cigars. Distinguishing between little cigars and traditional full-sized cigars, which are sometimes smoked as is and are sometimes emptied of tobacco and filled with marijuana (i.e., blunts), is also important. Some earlier studies differentiated cigars from blunts (1,2,26), but often the distinction between full-sized and little cigars is not made. If youth smokers consider little cigars to be neither cigarettes nor cigars (2,27) and a survey only asks about those 2 products, the survey may underestimate tobacco use.

Large recurring studies such as the National Youth Tobacco Survey (28) and the Youth Risk Behavior Survey (29) ask about cigars, cigarillos, and little cigars but not separately. Questions that lump these products together provide little information about the significance of little-cigar use among young African Americans (27). Furthermore, in neither survey do questions include examples of little-cigar brands. If some youth smokers are unaware that Black & Milds are little cigars, then the failure to provide brand examples may also lead to an underestimation of little-cigar use.

### Limitations

This study was limited to 3 focus groups of 21 college students at one historically black university, and the results may lack external validity. To what extent the focus groups shed light on the use of little cigars by African American young adults who are not in college, by black students at predominantly white colleges, or by college students who are not African American is not clear.

The focus group data were coded without qualitative data analysis software and therefore without the assistance such software provides in categorizing and reviewing qualitative data. However, the small number of participants made it easy to code, organize, and examine data by hand. Only the author analyzed the focus group data, so there was no check on the reliability of interpreting and categorizing text segments. This shortcoming is mitigated by 3 factors. First, the author was very familiar with the data, having been present during all focus groups. Second, the categories of interest were clearly but broadly defined, and the themes that emerged were not difficult to identify. Third, the author made no effort to quantify responses to particular questions beyond general groupings (e.g., a few, many, most). Misinterpretation or misclassification of a few statements should not alter basic conclusions of the analysis.

### Conclusions

Focus groups do not comprehensively define or describe an issue, but they provide information in participants' own words that deepen our understanding of the issue. In this study, focus groups were used to identify common habits and beliefs about little cigars that warrant further investigation and attention in the design of programs and materials to encourage tobacco prevention and cessation among African American college students. The results build on the findings of other research on cigar use by adolescents and young adults and extend our understanding of this smoking behavior.

However, given the preliminary nature of this study and its small number of participants, additional research on the use of little cigars by African American college students is needed to confirm and elucidate its findings. If verified, these results have several implications for tobacco control advocates at historically black colleges and universities and possibly at other campuses and other venues where African American young adults can be reached:

- Tobacco research targeting this audience should address the use of little cigars. Research on smoking is often limited to cigarettes, and when it does relate to cigars, it rarely considers them separate from full-sized cigars.
- Research on tobacco use should make clear distinctions among the various substances and products that can be smoked. If research does not differentiate between marijuana-filled "blunts" and cigars and if it does not specifically ask about little cigars and provide examples of little cigar brands, then tobacco use in this population may be miscalculated (1,2,27).
- Tobacco use prevention and cessation programs should address little cigars in materials, curricula, counter-advertising, and services. If tobacco use prevention programs do not plainly distinguish the substances and products that the target audience might use, these programs may confuse rather than enlighten.
- Tobacco programs should provide clear information about little cigars. Some little-cigar smokers are honestly confused about the health risks of using little cigars (1,2,5,26) and want the truth about using this product.
- Tobacco programs should debunk myths found in this and other (1,2,5,26) studies that:
  - Little cigars are not addictive.
  - Little cigars are a safe alternative to cigarettes.
  - Hyping reduces the health risk of little cigars.

This study hopefully will help define and promote additional research into the use of little cigars by African American college students and other college-aged African Americans. Research that targets this population and that addresses the use of little cigars in tobacco use prevention and cessation efforts needs to be addressed by the public health community. If it is not, then smoking behaviors that pose significant risks to African American young adults may be overlooked.
